# Computed tomography estimation of the prevalence of neuro-ophthalmic injuries in head trauma patients seen in a tertiary health facility in Ghana

**DOI:** 10.1016/j.heliyon.2020.e04200

**Published:** 2020-06-20

**Authors:** Philip N. Gorleku, Emmanuel K. Edzie, Klenam Dzefi-Tettey, Jacob Setorglo, Albert D. Piersson, Stephen Ocansey, Enyam K.A. Morny, Celso D.G. Armah

**Affiliations:** a Department of Medical Imaging, School of Medical Sciences, College of Health and Allied Sciences, University of Cape Coast, Cape Coast, Ghana; b P.M.B University of Cape Coast, Cape Coast, Ghana; c Department of Radiology, Korle-Bu Teaching Hospital, Accra, Ghana; d PMB, Accra, Ghana; e Department of Medical Biochemistry, School of Medical Sciences, College of Health and Allied Sciences, University of Cape Coast, Cape Coast, Ghana; f Department of Imaging Technology & Sonography, School of Allied Health Sciences, College of Health and Allied Sciences, University of Cape Coast, Cape Coast, Ghana; g Department of Optometry, School of Allied Health Sciences, College of Health and Allied Sciences, University of Cape Coast, Cape Coast, Ghana

**Keywords:** Neuroscience, Public health, Disability, Medical imaging, Radiology, Clinical research, Health promotion, Diagnostics, Road traffic accident, Traumatic brain injury, Neuro-ophthalmic injury, CT scan, Clinical audit, Vision loss

## Abstract

**Introduction:**

Prevalence of traumatic brain injury (TBI) is extremely high and potentially associated with severe incapacitating consequences. Literature reports that 90% of road traffic deaths and injuries including TBI occur in low and middle-income countries including Ghana. Computed Tomography (CT) scan is the imaging modality of choice for the initial assessment of the extent of head injury. Some Neuro-ophthalmic injuries (NOI) may sometimes be ambiguous and indistinct although a serious injury with potential damaging consequences. Data on the prevalence of NOI post trauma is non-existent in Ghana to inform policy. The onus therefore lies on the Radiologist who will review the head CT scan to be very meticulous not to miss any NOI if present. We therefore decided to diligently review a large cross-sectional retrospective post trauma head CT scans for occurrence of NOI.

**Objective:**

To determine the incidence of NOI secondary to head trauma and the possible loss of vision thereof in a retrospective study using patients' head CT scan data from a tertiary hospital's CT centre in Cape Coast, Ghana.

**Method:**

All head CT scans secondary to trauma for the period January 2016 to December 2018, were retrieved and carefully analysed. A total number of 1043 of head CT scan images were analyzed by Consultant Radiologists.

**Results:**

Results showed out of 1043 CT scans reviewed, 742 (71,1%) were males and 301 (28.9%) were females. A total of 609 (58.4%) out of the 1043 patients sustained NOIs of various anatomical types. More Males 398 (65.4%) sustained NOI than females 211 (34.6%). The incidence of NOI was more among the youth as majority 167 (27.4%) of the patients were within the 18–29 years followed by 30–39 years bracket of 148 (24.3%). Fourteen anatomical types of NOI were elicited and further analysis revealed, intra-ocular foreign body to be the highest 107 (17.6%) cases, orbital floor fractures injury was 92 (15.1%) cases, with globe rupture injury and intraocular hemorrhage recording 79 (13.0%) cases each. Optic nerve injury was the least revealing 7 (1.1%) cases. There was a relationship between the gender of patient and the propensity to sustain NOI as males were more disposed to NOI than females. Road traffic accident (RTA) was the main pervasive cause of TBI and this accounted for 71.9% of all cases, followed by fall from height 24%, and the least cause of TBI was ascribed to gunshot injury of 0.33%.

**Conclusion:**

Prevalence of NOI is high. Urgent measures must therefore be implemented to reduce the RTA menace in general and to mitigate the associated NOI and possible loss of vision thereof.

## 1 Introduction

Post traumatic head injury is the most prevalent emergency condition and one of the most common accident and emergency referrals accounting for 40–60% of trauma cases recorded at the emergency department in developing countries [1, 2]. A traumatic head injury is the main etiology of neuro-ophthalmic injuries. Eye Injuries are classified into blunt injuries, perforating injuries, injuries by foreign bodies and chemical injuries [3, 4, 5].

Some of the commonest neuro-ophthalmic injuries that maybe observed can be generally grouped into anterior chamber injuries, injuries to the lens, open-globe injuries, ocular detachments, intraorbital foreign bodies, carotid cavernous fistula, and optic nerve injuries. This may include optic nerve injury, corneal laceration, globe rupture, relative afferent pupillary defect (Deformation of Globe), visual acuity deterioration, abnormal eye movements, orbital floor fractures, partial and complete dislocation of the lens, vitreous detachment and retinal detachment [6, 7].

In a typical accident and emergency setting in Ghana, the initial assessment and triage of a patient with Traumatic Brain Injury (TBI) is usually done by the surgical team. It is therefore the prerogative of the emergency team on duty to request and refer the patient for one neuroimaging study or the other. After a complete imaging work up has been generated by a radiologist and in cases where an obvious eye injury has been established, intervention by an Ophthalmologist (who are very few in Ghana) is sought to assess the injury. The role of the Radiologist is key and therefore must pay attention to neuro-ophthalmic injuries. Prompt and proper management of TBI sequelae can significantly alter their course especially within 48 h of the injury. Neuroimaging techniques, which can determine the presence or otherwise the extent of injury and guide surgical planning and interventions, play particularly important role in the emergency management of TBI. The prognosis of the injury and the implications for vision especially for globe injuries can then be elicited [7]. It has been estimated that 40% or more of monocular vision loss in the United States is secondary to trauma [8].

Patients with head injury undergo one or more imaging examinations in the form of plain radiography (e.g. skull X-ray), ultrasonography, computed tomography (CT), and/or magnetic resonance imaging (MRI). Plain radiography has a high sensitivity (up to 78%) for fractures, but very low sensitivity for soft-tissue injuries to the orbital contents [9] and are therefore limited when assessing serious injury to the brain and orbit. The use of ultrasound for evaluating the globe and its contents can be especially useful, however, it is contraindicated if a ruptured globe is suspected. MRI is the examination of choice for superior soft-tissue discrimination of intracranial anatomy. However, it cannot be used if there is the presence of a metallic intra-orbital foreign body.

MRI may be difficult to perform in emergency situations because it is time-consuming before the images are generated; it is not easily accessible in Ghana [11]; using MR imaging for the initial evaluation of an orbital trauma is not recommended, although it may be very useful once the initial imaging has been performed. However, there are circumstances where CT may be immensely helpful, in addition to MR, such as in demonstrating calcification and bony pathology.

Computed Tomography is the modality of choice for the initial evaluation of a traumatic injury to the globe, especially when intraocular or intraorbital foreign body is suspected. CT has also been shown to be more accurate than radiography in detecting fractures. When fractures are present, three-dimensional reconstruction of the CT images is a useful tool to guide surgical treatment [10].

CT scan is superior in detecting vitreous hemorrhage and lens dislocation in head injury patients usually within the first 24 h and requires less time to produce diagnostic results [12]. Orbital and head CT scans are widely available, non-complex to perform, relatively inexpensive, and the images can be obtained very promptly. CT scan can effectively diagnose various types of neuro-ophthalmic injuries and is therefore the imaging modality of choice to evaluate traumatic orbital conditions. Selective application of contrast agents improves the sensitivity and specificity of CT scan interpretation [13]. However, contrast material for CT scanning is iodinated contrast to which patients can sometimes have adverse reactions. Prior allergic reaction to iodinated contrast or a history of renal failure may be contraindications to using contrast in CT [14]. In fact, the non-contrast study is superior for assessing the hyper density of acute blood therefore in cases of trauma (e.g. orbital, facial or skull base fracture, orbital or intracranial foreign body, or traumatic optic neuropathy), contrast material adds little to the examination.

Neuro-ophthalmic injury (NOI) secondary to trauma is a common manifestation and a major cause of blindness and vision deficits [15]. Some NOIs may sometimes be ambiguous and indistinct albeit a serious injury with potential damaging consequences.

However, no data exists on prevalence of NOIs in Ghana and therefore the magnitude of incidence of NOIs post trauma has not been well appreciated in order to inform strategy as to the degree of requirement for urgent Ophthalmological intervention which is critical to prevent permanent loss of vision.

The responsibility therefore lies on the Radiologist who maybe the first to diagnose a neuro-ophthalmic injury if any, to diligently pay specific attention to these otherwise serious injuries that may not necessarily be obvious.

We therefore decided to conduct a large cross-sectional retrospective study of all head CT scan cases secondary to trauma in which we diligently reviewed all the CT scan images of patients seen at a tertiary hospital's CT scan centre in Ghana for the occurrence of Neuro-ophthalmic injury. In this paper, we report CT findings of Neuro-ophthalmic injury prevalence from Traumatic head injury.

## 2 Methodology

### 2.1 Study area

The study was conducted at the Cape Coast Teaching hospital which is one of the four teaching hospitals in Ghana. Cape Coast is a Metropolis, the administrative capital of the Central Region of Ghana. The hospital provides health care services to patients from the metropolis and indeed from the entire central region and beyond. The central region is one of the 10 regions in Ghana and covers an area of 9,826 square kilometers and is the eighth ranked region by land size in Ghana. The population of the region according to the 2010 population and housing census stands at 2,201,863 with 1,050,112 males and 1,151,752 females.

### 2.2 Study population

The study involved review of 1,043 head CT scan images of patients who had undergone head CT and met our inclusion criteria between the period of January 2016 to December 2018.

### 2.3 Study design

This was a retrospective study. All head CT scan data of patients who had undergone CT scan secondary to head trauma were included. The CT scans were performed on a Toshiba Aquilion 16- CT-scanner. All scans meeting our study criteria were diligently reviewed by consultant radiologists by systematically and progressing from anterior to posterior with the intension to first look for external soft tissue changes then assessing the anterior chamber, position of the lens, globe and posterior segment, bony orbit, foreign bodies, ocular vessels and optic nerve as suggested by Kubal [16]. The age, gender, source of injury, the presence and type of neuro-ophthalmic injury (NOI) if present were evaluated.

### 2.4 Inclusion criteria

All head CT scans secondary to trauma from January 2016 to December 2018 were retrieved for review.

### 2.5 Exclusion criteria

All other head CT scans not related to trauma were excluded.

Some of the CT images were not clear enough and of poor quality for the radiologist to make a conclusive diagnosis, so such images were excluded from the study.

### 2.6 Data collection

The collection of data was based on primary data from the review of the head CT scans of patients that met our inclusion criteria.

### 2.7 Data analysis

The data obtained was entered and analyzed using the Statistical Package for Social Science (SPSS v.23.0 software). Proportions were presented for categorical variables using frequencies, percentages, and Chi-square where appropriate. A p value of <0.05 was considered statistically significant.

### 2.8 Ethical clearance

Ethical clearance and permission to undertake the research was sought from the Cape Coast Teaching Hospital Ethical Review Committee (Reference number: CCTHERC/EC/2016/05). Data security and confidentiality was assured as names and other personal information were not included in the study.

#### 2.8.1 Limitations of the study

• There was no direct link between the CT scan center and the outpatient department. Thus, patient's information such as, short history, presenting complains, occupation, presenting vision and visual outcome could not be obtained.
• All the causes of head trauma related to the road was lumped together as RTA. We could not therefore isolate cases of pedestrian knock downs and motorbike accidents.

## 3 Results

Between 1st January 2016 to 31^st^ December 2018, a total of 1,043 patients had head CT examinations secondary to trauma of which 742 (71,1%) were males and 301 (28.9%) females. A review of the 1,043 head CT scans elicited 609 (58.4%) various types of neuro-ophthalmic injuries (NOI).

**Figure 1:**
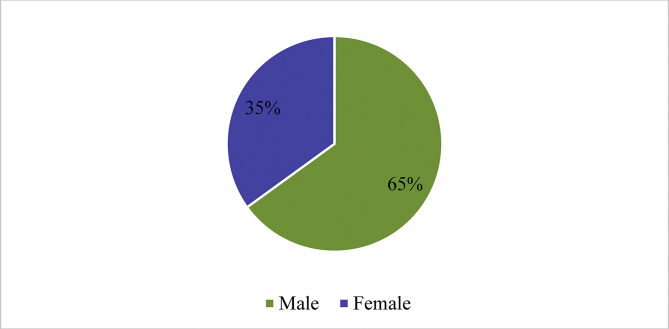
Gender distribution of patients who sustained NOI between 2016 to 2018.

The sex distribution of the 609 patients who sustained NOI was, 398 (65%) males and 211 (35%) females as illustrated in Figure 1.

**Figure 2:**
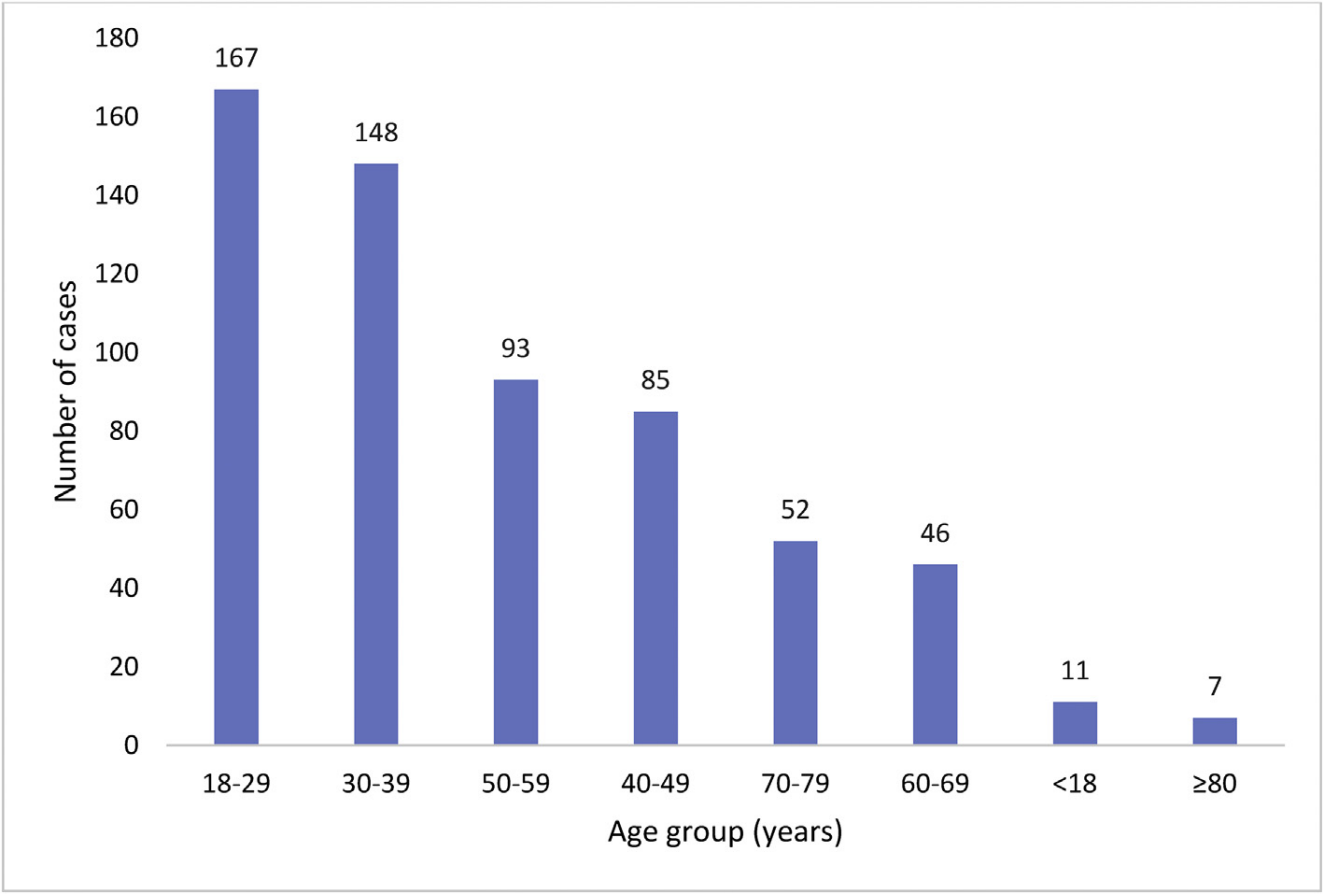
Age distribution of patients and incidence of injury.

Analysis of the age distribution of the NOI patients showed, 11 (1.8%) patients below the age of 18 years old. Majority 167 (27.4%) of the patients who sustained NOI were aged 18–29 years. A further 148 (24.3%) patients were within the 30–39 years bracket. The age range of 40–49 years had 85 (14.0%) patients, while the 50-59-year age group was 93 (15.3%), and the 60-69-year group was 46 (7.6%) cases. The age group of 70–79 years was 52 (8.5%) and those of 80 years and above were seven representing 1.1% and this is illustrated in Figure 2.

Further analysis of the scans elicited a total of 14 types of NOI of various anatomical location as shown in Table 1.

**Table 1:** Anatomical distribution of Neuro-Ophthalmic Injuries.

Type of injuries	No. of Patients	Percentage (%)
Intra Ocular Foreign Body	107	17.6
Orbital floor fractures	92	15.1
Globe rupture	79	13
Intraocular Haemorrhage	79	13
Displaced Cornea & Anterior Sclera	46	7.6
Hemorrhagic choroidal detachment	44	7.2
Corneal laceration	37	6.1
Carotid cavernous fistula	26	4.3
Deformation of Globe	22	3.6
Partial and Total Lens Dislocation	21	3.4
Intraocular Air	19	3.1
Vitreous and Retinal Detachment	16	2.6
Entrapment of extra ocular muscle	14	2.3
Optic Nerve Injury	7	1.1
Total	609	100

**Figure 3:**
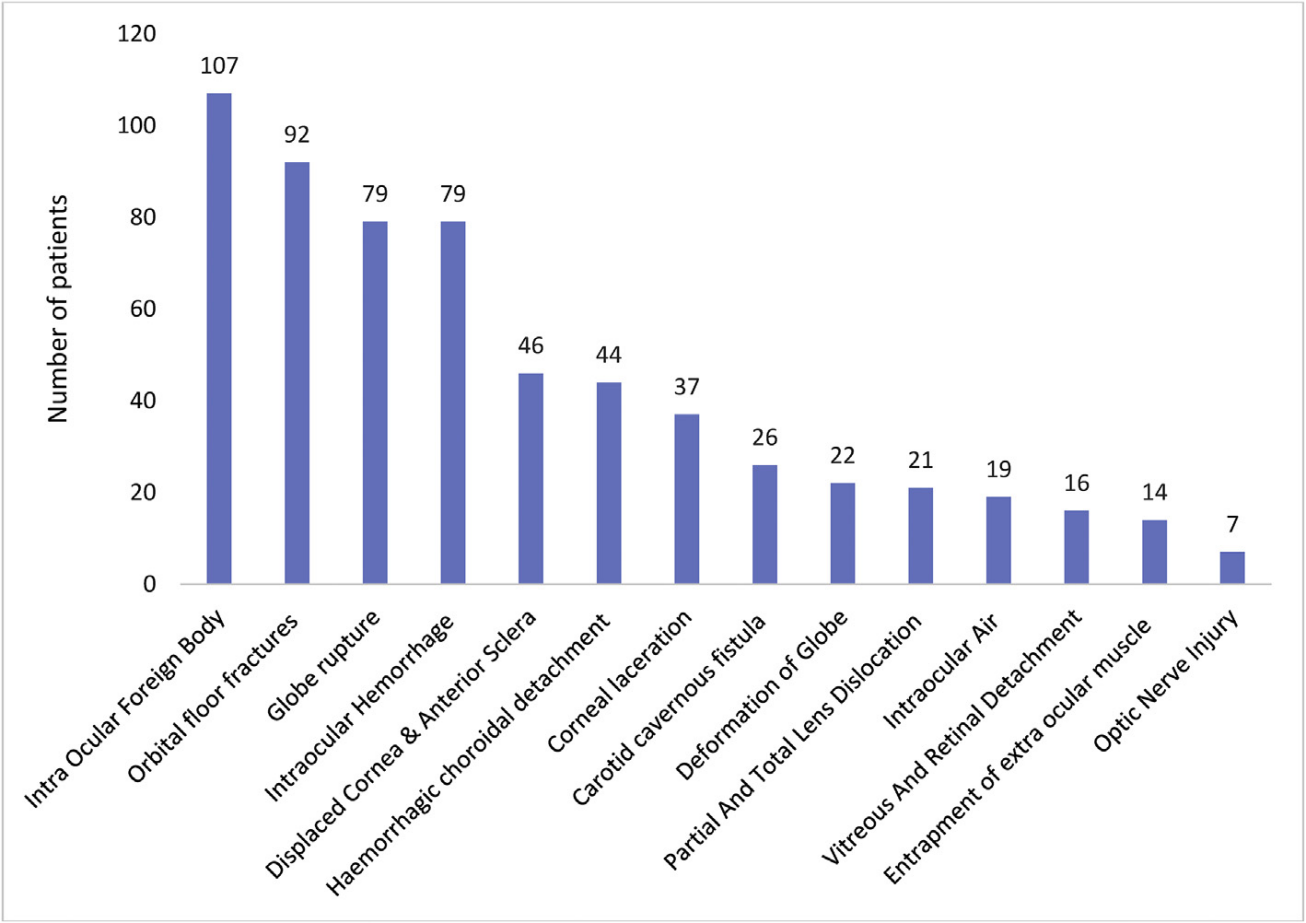
A bar graph showing the Distribution of Neuro-Ophthalmic Injuries.

Table 1 shows the types and anatomical distribution of the various cases of NOI. The inference can be made that, of the 609 patients who sustained NOI, majority 107 (17.6%) had Intra Ocular Foreign Body followed by Orbital floor fractures with 92 (15.1%) cases. The least number of cases was optic nerve injury which was seven representing 1.1% of all the NOI elicited. This is illustrated in Figure 3.

A chi-square test was performed to ascertain the relationship if any, of sex and the type of NOI sustained using α-level of 0.05. There is an indication that there is a relationship between sex and the type of injuries sustained since p-value (p < 0.001) is less than α-level (0.05) that is p< α. Therefore, there is statistically significant (Chi-square = 60.125, df-13, p < 0.001) relationship of sex and type of injury sustained. This is an indication that males are predisposed to various types of Neuro-Ophthalmic injuries than females as shown in Table 2.

**Table 2:** Distribution of neuro-ophthalmic injuries based on gender.

Type of Injury	Observation		Number and percentages of	
	Number	Percentage	Males	Females
Intra ocular foreign body	107	18	57 (53.0%)	50 (47.0%)
Orbital floor fractures	92	15	50 (54.3%)	42 (45.7)
Globe rupture	79	13	49 (62.0%)	30 (38.0%)
Intraocular Hemorrhage	79	13	55 (69.6%)	24 (30.4%)
Displaced cornea & anterior sclera	46	8	42 (91.0%)	4 (9.0%)
Haemorrhagic choroidal detachment	44	7	31 (70.5%)	13 (29.5%)
Corneal Laceration	37	6	28 (76.0%)	9 (24.0%)
Deformation of Globe	22	4	19 (86.4%)	3 (13.6%)
Carotid Cavernous Fistula	26	4	24 (92.3%)	2 (7.7%)
Partial and Total Lens Dislocation	21	3	9 (43.0%)	12 (57.0%)
Vitreous and Retinal Detachment	16	3	6 (37.6%)	10 (62.5%)
Intraocular Air	19	3	10 (52.6%)	9 (47.4%)
Entrapment of extra ocular muscle	14	2	11 (78.6%)	3 (21.4%)
Optic nerve injury	7	1	5 (71.0%)	2 (29.0%)
Total	609	100	398	211

Table 3 shows a cross-tabulation of the prevalence of the various Neuro-Ophthalmic injuries compared to the age of the patients. The result was statistically significant (Chi-square = 515, df-91, p < 0.001). It showed that out of a total of 107 (17.6%) patients who had intra-ocular foreign body, 37 and 30 which forms majority of this group were youthful, within the ages of 18–29 and 30–39 respectively. This is followed by those who had orbital floor fractures 92 (15.1%) with 29 and 25 patients within the ages of 18–29 and 30–39 respectively which constituted most of this group. Globe rupture and intraocular hemorrhage both recorded 79 (13.0%) each. In the case of globe rupture most patients were within the age group of 18–29 (22) followed by 50–59 (21), then 40–49 (20) with no patient below 18 and between 70-79 years whereas for intraocular hemorrhage, most of the patients fell within the age group of 30–39. From Table 3, the youth dominated in most of the patients who sustained neuro-ophthalmic complications secondary to head injuries except in the case of Corneal Laceration which has majority 18 out of the 37 cases were within the age of 70–79. The age group of 60–69 years recorded highest in partial and total lens dislocation with 8 out of a total of 21. It can also be inferred that patients at the age of 80 and above sustained more intraocular hemorrhage than any other form of injuries, there was a total of five out of seven.

**Table 3:** Distribution of the types of neuro-ophthalmic injuries Based on age of patients.

Type of neuro-ophthalmic injury	Age group (years)								
	0–17	18–29	30–39	40–49	50–59	60–69	70–79	80–89	Total
Intra ocular foreign body	3	37	30	1	13	14	9	0	107
Orbital Floor Fractures	2	29	25	17	8	7	4	0	92
Globe rupture	0	22	12	20	21	3	0	1	79
Intraocular Haemorrhage	1	10	33	15	10	4	2	4	79
Displaced cornea & anterior sclera	2	16	5	2	15	3	3	0	46
Haemorrhagic choroidal detachment	3	29	2	3	2	2	3	0	44
Corneal laceration	0	2	4	5	4	3	18	1	37
Carotid cavernous fistula	0	1	22	1	1	0	1	0	26
Deformation of globe	0	12	1	0	6	0	3	0	22
Partial and total lens dislocation	0	1	0	7	2	8	3	0	21
Intraocular Air	0	2	2	3	7	2	3	0	19
Vitreous and retinal detachment	0	0	4	8	2	0	2	0	16
Entrapment of extra ocular muscle	0	3	5	2	2	0	1	1	14
Optic nerve injury	0	0	3	4	0	0	0	0	7
Total	11	164	148	88	93	46	52	7	609
Percentage	1.8	27	24.3	14.4	15.3	7.6	8.5	1.1	100

**Figure 4:**
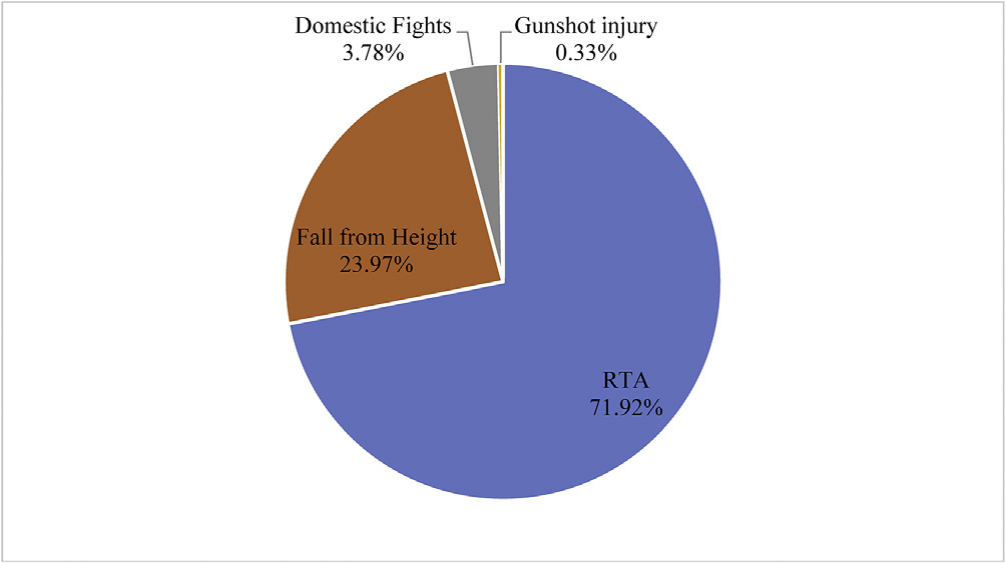
Causes of head injury.

The causes of head injury observed in the 609 patients with neuro-ophthalmic injuries were grouped into RTAs, fall from heights, domestic fights, and gunshot injury. RTA was the most pervasive cause of head injury with 438 (71.9%) patients, followed by domestic fights 23 (3.8%) cases, and gunshot injury was the least cause with 2 (0.33%) cases as illustrated in Figure 4**.**

## 4 Discussion

This study documents for the first time and evaluates the prevalence in Ghana, neuro-ophthalmic injuries (NOI) secondary to head trauma, the possibility of vision loss thereof as diagnosed by CT scan in a tertiary hospital. Some NOI may sometimes be ambiguous and indistinct though a serious injury with potential damaging consequences.

The globe and orbit form an exceedingly small portion of the body but trauma to this region assumes a critical importance due to the high value we place on vision. The loss of vision is a catastrophe, and this need not be over emphasized. The responsibility therefore lies on the Specialist Radiologist to meticulously review a post traumatic head CT scans for possible presence of NOI if any. This study therefore revealed that NOI abounds and can be adequately diagnosed by CT scan which was the imaging method of choice.

Several researches have been conducted to ascertain the efficacy of the application of CT in the diagnosis and management of post trauma ocular injuries. From the available literature and the results obtained from our study, it confirmed the role of CT in the diagnosis of post traumatic NOI is unequivocal and our findings is consistent with previous studies. Chaudhary et al, [12] conducted a retrospective study on ‘The Role of Computed Tomography in Predicting Visual Outcome’ in ocular trauma patients consisting of soldiers with facial and/or suspected ocular injuries. A total of 80 CT images of eyes with varying blast injuries were studied and assessed by military consultant radiologists. The results in comparison with actual clinical findings showed that out of the 40 patients studied, 11 had unilateral injuries while 16 had bilateral injuries. No pathological findings were reported in 37 of the 80 eyes and adnexa. Where no pathological findings were reported, the eyes and adnexa were all found to be either intact; or had relatively minor closed globe injuries, such as corneal abrasion or optic nerve injury, that did not require surgical intervention. All foreign bodies were successfully diagnosed by CT scanning. Extra ocular foreign bodies were found in 3 out of 80 eyes (4%) while intraocular foreign bodies (IOFB) was found in 14 (17.5%). All 18 (22.5%) penetrating injuries were diagnosed on CT, though 4 eyes did not have direct CT imaging evidence of a corneal or scleral (eye wall) defect or a collapsed globe. Intraocular air was present in 8 (57%) of 14 eyes with an IOFB. Of the 18 eyes with a corneal or scleral defect, 12 (66.7%) had intraocular air present on CT scan, and 6 (33.3%) did not. The paper concluded that CT scan is particularly useful in the initial diagnosis of globe injuries and in the absence of clinical information, CT imaging sensitivity and specificity has been reported as 75% and 93% respectively for the prediction of open-globe injury.

In another study, Yuan et al. [17], evaluated the CT characteristics of Globe Rupture. The medical records of patients seen in the emergency department with blunt, penetrating, or explosive orbit injury with no history of eye disease were retrospectively reviewed. A total of 75 patients (76 injured globes) were included. CT examinations were reviewed by two experienced radiologists without knowledge of ophthalmologic findings, original orbital CT images, or surgical outcomes. The study showed a good interrater agreement between the two radiologists (kappa value range, 0.63–0.96). The average sensitivity, specificity, positive predictive value (PPV), negative predictive value (NPV), and accuracy of CT for the detection of globe rupture based on readings by the two radiologists were 76%, 85%, 80%, 82%, and 81%, respectively.

However, CT scan can always be complimented by MR imaging when the patient is stable in order not to miss any pathology. It is important also that eye care practitioners collaborate very closely with Radiologists and become familiar with ocular imaging and the various characteristic signs of serious ocular injuries in order to arrive at an accurate diagnosis and commence treatment or refer appropriately.

Our study resulted in the diagnosis of 14 vision threatening neuro-ophthalmic injuries. The manifestation of post traumatic NOI was high (58.4%) as was demonstrated by our study and this is very consistent with other studies [2, 7]. A research conducted in Australia reported that 16% of major trauma patients had ocular or orbital trauma whereas 55% of patients with facial injuries had ocular or orbital injuries [16]. In a related study of pediatric patients with major trauma in the United States, 8% of the patients sustained an ocular injury [16, 18]. Ocular injuries are key to the overall patient outcome, for example, for patients diagnosed of ruptured globe, 29% underwent enucleation with additional 29% having poor visual acuity or no light perception [16].

From our study, out of the total 609 patients with ocular involvement, 398 (65%) were males and 211 (35%) were females as (a ratio of almost 2:1, male: female). The age range of 18–59 accounted for a total of 493 (81%) of patients which infers the significant numbers of potential debilitating injuries sustained by the working class and this is very worrisome [19].

Intra ocular foreign body (IOFB) had the highest (107, 17.6%) prevalence. This could be attributed to the fact that modern day cars are equipped with airbags which produces a blast effect in an attempt to save the driver when there is an accident [21, 22]. Timely identification and removal of IOFB reduces the risk of endophthalmitis [20]. Orbital floor fractures were (92, 15.1%) and has been one of the most common ocular injuries whenever there is blow out trauma to the head. The inferior wall is the most frequently affected area in blow out traumas because it is the weakest portion of the entire orbit and theories have been developed to explain this concept [21]. Surgical repair and management require accurate imaging and topographical diagnosis of the orbital floor fracture, the surgeon must have a clear picture of how the bones are arranged in order to attempt surgery. The CT image provides a clear picture of the orbital floor which helps the surgeon to see if there is any incarceration or entrapment of soft tissue related to the orbit within the adjacent sinus [22].

Globe rupture (79, 13.0 %) usually happens at the point of extra ocular muscle insertion where the sclera is thinnest. Globe rupture can be diagnosed clinically by just looking at the intraocular structures. However, in a setting where this is not possible, CT scan is able to accurately diagnose globe rupture and the three most common diagnostic features are globe deformity or wall irregularity, eyelid hematoma or swelling and intraocular hemorrhage [17]. However, in our study the strongest indicative feature for globe rupture was globe deformity. Thus, the diagnosis of intraocular hemorrhage did not have the other features of globe rupture, especially globe deformity. All these characteristics were clearly identified on the CT scan images in our study.

All the other NOI need urgent surgical intervention in order to save eyesight. However, ophthalmologists are only found in the major hospitals in Ghana. Thus, the optometrist plays an important role by accurately diagnosing the condition, giving an initial or emergency care before referring to the ophthalmologist for surgery. The causes of head trauma from our study can be categorized into four groups; Road Traffic Accident (RTA) was the most predominant and most pervasive with 438 (71.9%), followed by fall from height 146 (24%), industrial and domestic accidents was 23 (37.8%) and the least was gunshot injury which was two, representing 0.33% cases. The inference therefore can be made that, the use of guns in Ghana is extremely limited. The RTA situation in Ghana is very appalling and has become a serious public health treat if urgent and drastic measures are not implemented. The socio-economic implications are very catastrophic. Data from Government stipulates that, the country spends 230 million United States Dollars annually treating injuries and traffic fatalities [23]. Furthermore, statistics from the Motor Traffic and Transport Department of the Ghana Police Service indicates that road accidents in the country continue to increase with many more people dying from road crashes. More men than women are involved in road crashes and the RTA trend is exacerbating. Data from the Motor Traffic and Transport Department (MTTD) in Ghana shows a death toll of 696 in the first quarter of 2019, a 17.57% increase for the same period in 2018. There were 2,341 deaths in 2018 from RTA representing an increase of 12.76% compared to 2017. Pedestrians are also knocked down rampantly. Out of the 2,341 (1,796 males and 545 females) killed in 2018, 795 (34.0%) were pedestrians [24]. Globally, according to the World Health Organization (WHO), every year, 1.25 million people lose their life prematurely due to road traffic accidents. Additionally, 90% of all RTA and the resulting deaths and injuries occur in low and middle-income countries with highest rates in Africa, including Ghana. Among some of the causes of RTAs are poor driving skills, drivers talking on mobile phones while driving, gross indiscipline and broken-down vehicles on roadsides etc. Furthermore, one can allude to factors like the use of worn-out second-hand tires, overloading, neglecting traffic regulations, as well as lack of road markings, signs and maintenance can be cited.

The National Road Safety Commission of Ghana has bemoaned the increase in the number of road crashes as it causes innocent people to be compulsorily physically challenged and put others in the state of perpetual poverty and premature death. There is therefore the need to take drastic measures to reduce the numbers of RTA, unnecessary deaths and profoundly serious and debilitating injuries including neuro-ophthalmic injuries.

It is our earnest wish that, planners, policy makers, and medical practitioners will initiate and implement concerted joint action to eliminate this unacceptable and needless health risk.

## 5 Conclusion and recommendations

Radiologists and eye specialists in Ghana are few [25, 26] and there is high pressure to evaluate many CT scan images of head trauma patients. From our study and experience, body imaging covering multiple organ systems in severely traumatized patients can be very challenging and therefore, a comprehensive checklist is useful for evaluating the orbit and its contents; first, evaluate the bony orbit for fractures and take note of any herniation of orbital contents. Pay attention to the orbital apex where a tiny fracture may even be a sign for emergent surgery, evaluate the anterior chamber. Increased attenuation suggests an optic nerve injury. Decreased depth gives a clue to either a corneal laceration or anterior subluxation of the lens. On the other hand, increased depth is linked to open-globe injuries; assess the position of the lens. Also, bear in mind that the lens may be displaced either anteriorly or posteriorly which may also be completely or partially; evaluate the posterior segment of the globe. Look for abnormal fluid collections or bleeds. Try to localize the fluid collection not forgetting the characteristic shape of fluid collections in a choroidal or retinal detachment. Also, look for signs of radiopaque or radiolucent foreign bodies. Wooden foreign bodies can mimic air on CT scans; and in conclusion assess the ophthalmic veins as well as the optic nerve complex. For dilated ophthalmic veins, check for other signs of carotid cavernous fistula. In penetrating traumas for example, the optic nerve may be transected. In blunt traumas, orbital apex is the key area to evaluate [16].

Documentation at the CT scan department on trauma patients must be more detailed and should include a brief history about the manner the injury was sustained. Pedestrians and motorbike riders who are knocked down must be differentiated and not labelled as general RTA cases as we observed.

Road traffic accidents cost most countries 3% of their gross domestic product. The WHO estimates that RTA will be the third leading cause of death by 2020 if measures are not put in place to curb the situation. Road traffic accident is a public health concern and a shared responsibility and therefore, while the number of road accidents keeps increasing in Ghana, preemptive measures must be put in place by all stakeholders to reduce the menace. Formulation of road safety policies and enforcement of road safety rules and regulations must be targeted at reducing road traffic accidents by creating low-speed zones in urban settings, setting speed limits according to road type, enforcing the wearing of requisite helmets and incessant use of seat belts etcetera.

The National Road Safety Commission (NRSC) in collaboration with other stakeholders should embark on road safety campaigns to educate all road users on road safety issues and the need to abide by all road regulations. There should be continual effort by the Ghana Highways Authority together with the Department of Urban Roads to maintain and rehabilitate all bad roads. The Ghana Police Service should be well-equipped and motivated to check, arrest and prosecute all careless drivers and passengers who flout road regulations.

Road accidents can be controlled and therefore, all and sundry must join the campaign to reduce the carnage on our roads. This will see a drastic reduction in the prevalence of neuro-ophthalmic injuries and the accompanying complications thereof.

**What this Study Adds**

• The demonstration of the prevalence of post traumatic neuro-ophthalmic injury, the types, and the socio-economic impact.
• Highlighted the importance of Radiologists to diligently assess and evaluate the orbitals for presence of neuro-ophthalmic injuries for head CT scan post trauma.
• Highlighted the importance of the close collaboration between the Optometrist and Ophthalmologist with the Radiologist.
• The Study highlighted the appalling RTA situation in Ghana and must be treated as a serious public health disaster.

## Data availability statement

Anonymised data relied on for this study may be released upon application to the Head of Department of Radiology, Cape Coast Teaching Hospital, after approval by the Ethical Review Committee of the Cape Coast Teaching Hospital, Ghana. Email: info@ccthghana.com.

## Declarations

### Author contribution statement

Philip N. Gorleku, Emmanuel K. Edzie: Conceived and designed the experiments; Analyzed and interpreted the data; Wrote the paper.

Klenam Dzefi-Tettey: Analyzed and interpreted the data.

Jacob Setorglo: Contributed reagents, materials, analysis tools or data; Wrote the paper.

Albert D. Piersson, Stephen Ocansey: Performed the experiments; Contributed reagents, materials, analysis tools or data; Enyam K. A. Morny: Conceived and designed the experiments; Contributed reagents, materials, analysis tools or data.

Celso D. G. Armah: Performed the experiments.

### Funding statement

This research did not receive any specific grant from funding agencies in the public, commercial, or not-for-profit sectors.

### Competing interest statement

The authors declare no conflict of interest.

### Additional information

No additional information is available for this paper.
